# Determinants of health insurance coverage among women in Mauritania: a multilevel analysis

**DOI:** 10.1186/s12889-024-17691-y

**Published:** 2024-01-19

**Authors:** Robert Kokou Dowou, Gideon Awenabisa Atanuriba, Leticia Akua Adzigbli, Samuel Kwaku Balame, Issifu Tahidu, Juliet Aggrey-Korsah, Richard Gyan Aboagye

**Affiliations:** 1 Department of Epidemiology and Biostatistics, Fred N. Binka School of Public Health, University of Health and Allied Sciences, Hohoe, Ghana; 2 Department of Quality Improvement, Northern Regional Hospital, Tamale, Ghana; 3 Department of Academics, School of Hygiene, Ho, Ghana; 4 Department of Public Health, School of Hygiene, Tamale, Ghana; 5 Department of Family and Community Health, Fred N. Binka School of Public Health, University of Health and Allied Sciences, Hohoe, Ghana

**Keywords:** Health insurance coverage, Women, Mauritania, Demographic and Health Survey

## Abstract

**Background:**

Health insurance has been documented as one of the primary methods of financing healthcare for Sustainable Development Goals (SDGs) by 2030. Yet, there is a dearth of evidence on the determinants of health insurance coverage among women in Mauritania. We examine the factors associated with health insurance coverage among women in Mauritania using a nationally representative survey dataset.

**Methods:**

We analyzed secondary data from the 2019–2021 Mauritania Demographic and Health Survey. A weighted sample of 15,714 women of reproductive age (15–49 years) was included in the study. Multilevel regression analysis was used to examined the factors associated with health insurance coverage. The results were presented using an adjusted odds ratio (aOR) with a 95% confidence interval (CI).

**Results:**

The coverage of health insurance among women was 8.7%. The majority of the women subscribed to social security health insurance (7.6%). Women aged 35 years and above [aOR = 1.54; 95% CI = 1.24, 1.92] were more likely to be covered by health insurance relative to those aged 15–24. The likelihood of being covered by health insurance increased with increasing level of education with the highest odds among women with higher education [aOR = 6.09; 95% CI = 3.93, 9.42]. Women in the richest wealth index households [aOR = 22.12; 95% CI = 9.52, 51.41] and those with grand parity [aOR = 2.16; 95% CI = 1.62, 2.87] had the highest odds of being covered by health insurance. Women who were working, those who watched television, and those who used the internet were more likely to be covered by health insurance relative to their counterparts who were not working, those who did not watch television, and those who did not use the internet. Women residing in Tiris zemour et Inchiri [aOR = 3.60; 95%CI = 1.60, 8.10], Tagant (aOR = 3.74; 95% CI = 1.61, 8.68], and Adrar [aOR = 2.76; 95% CI = 1.36, 5.61] regions were more likely to be covered by health insurance compared with those from Hodh Echargui.

**Conclusion:**

Health insurance coverage among the women in our study was low. Achieving the SDG targets of ensuring universal health coverage and lowering maternal mortality to less than 70 deaths per 100,000 live births requires the implementation of interventions to increase health insurance coverage, taking into consideration the identified factors in the study. We recommend effective public education and awareness creation on the importance of being covered by health insurance by leveraging television and internet platforms. Also, interventions to increase health insurance coverage should consider younger women and those in rural areas.

## Background

Globally, health insurance has been documented as one of the primary methods of financing healthcare for Sustainable Development Goals (SDGs) by 2030. Countries across the developing world are poised to achieve the SDGs agenda, which they subscribed to by 2030 [1]. Universal Health Coverage (UHC) is one of the key means of achieving the SDG target 3.8 by these countries. UHC is when all people have equal access to health services without financial constraints. UHC is therefore targeted at protecting people from the financial burden of paying for health services out of pocket [2].

Notwithstanding the improvement in the availability of modern healthcare in sub-Saharan Africa (SSA), access to health services by the population remained poor mainly due to the weak health sector financing system and the inability of people to afford it [3–5]. Without a well-functioning healthcare financing system, universal access to health services cannot be achieved for the majority of the population [6]. The dependence on out-of-pocket expenditure to finance health care is not an uncommon feature in the health system of many low-and middle-income countries (LMICs) [7].

In SSA, access to health care remains limited because of financial constraints. Out-of-pocket payments are among the main factors which prevent the majority of the people in these countries from accessing timely and adequate health care [2]. This comes against the backdrop of the World Health Organization’s (WHO) recommendation that countries develop a financing system so that all people have access to services without any financial difficulties [8]. Households without adequate financial protection for healthcare services face a high risk of sustaining large unanticipated healthcare expenditures should they decide to utilize healthcare services. These unexpected expenditures sometimes push households to spend substantial proportions of their disposable income leading them to indebtedness and reduction in living standards [9, 10].

One major approach to mitigating out-of-pocket payments and improving people’s access to health care with efforts to achieve UHC in SSA is health insurance [11]. Effective health insurance coverage affects households by leading to better healthcare service utilization, especially among women who constantly need maternal and child health services as it reduces out-of-pocket health expenses [12, 13]. Inadequate or no health insurance coverage creates financial barriers to healthcare services, especially for vulnerable populations [13].

Previous studies conducted in LMICs have stipulated that health insurance coverage among women remains low and this could have dire implications on the extent of access to health services, especially among the socioeconomically disadvantaged populace. For instance, a study conducted in SSA reported the overall health insurance coverage to be 8.5%, ranging from 0.9% in Chad to 62.4% in Ghana [14]. Among urban women in SSA, the pooled coverage of health insurance was 40.6% [15]. However, none of these studies included Mauritania, indicating a gap requiring urgent research.

Numerous studies have examined the determinants of health insurance subscription and identified socio-demographic factors such as sex, age, economic factors, place of residence, household size, and behavioral factors to be associated with health insurance subscription [16–18]. Despite the aforementioned factors influencing health insurance coverage, evidence of such determinants in Mauritania remains limited. Our study sought to examine the determinants of health insurance coverage among women in Mauritania. This study could be relevant to policymakers and intervention or program planners, as it will deepen their understanding of the factors that influence women’s decision to subscribe to health insurance. This knowledge could help policymakers decide on the best strategies to adopt and implement that would increase health insurance coverage among women in Mauritania.

## Materials and methods

### Data source and study design

We analyzed secondary data from the 2019–2021 Mauritania Demographic and Health Survey (DHS). The data was extracted from the individual recode file (women's file). DHS is a nationally representative survey conducted every five years in over 90 low-and middle-income countries worldwide [19]. The Mauritanian DHS employed a cross-sectional design, with respondents sampled using a two-staged cluster sampling technique [20, 21]. The detailed sampling methodology has been published elsewhere in the literature [20, 21]. Pretested and validated questionnaires were used to collect data from the respondents [19]. Trained data collectors were used for the survey. In our study, a weighted sample of 15,714 women aged 15–49 with complete observations on variables of interest was analyzed. The dataset used can be accessed via https://dhsprogram.com/data/dataset/Mauritania_Standard-DHS_2020.cfm?flag=1. We drafted this paper per the Strengthening Reporting of Observational Studies in Epidemiology (STROBE) guidelines [22].

### Variables

Health insurance coverage was the outcome variable in our study. This variable was measured in the DHS using the question: *Are you covered by any health insurance*?”. In the DHS, the response options to the question were “0 = no” and “1 = yes”. We utilized these definite responses in our final analysis. Previous studies using the DHS employed the same categorization [11, 14, 15, 23, 24].

Based on the review of pertinent literature [11, 14, 15, 23, 24], we included thirteen explanatory variables in our study. Also, these variables were available in the DHS dataset. The variables include women’s age, level of education, marital status, working status, parity, watch television, listen to radio, read newspapers or magazines, internet usage, wealth index, sex of household head, place of residence, and region. The women were asked to indicate their current age in a discrete form which we categorized into 15–24; 25–34; and 35 years and above. Level of education represented the highest educational attainment of the women which was coded in the DHS as no education, primary, secondary, and higher. For working status, the women who were working at the time of the survey were coded as working (yes), otherwise coded as not working (no). Current marital status was coded as never in union, married, divorced, and widowed. With parity, the women were asked to indicate the number of children they had given birth to. Those without children were categorized as nulliparity. Those with one child, two to four children, and five or more were classified as primiparity, multiparity, and grandparity, respectively. In the DHS, each frequency of reading newspapers/magazines, frequency of listening to the radio, and frequency of watching television was categorized into “not at all, less than once a week, and at least once a week”. In this study, those whose response option was not at all were categorized as ‘no’ and the remaining response options were merged and coded to form the ‘yes’ category. The wealth index was measured mainly based on component rankings generated through principal component analysis on ownership of family assets, for example, supply of drinking water, kind of toilet facility, sort of cooking fuel, and possession of television and fridge. Based on individual rankings, the households were grouped into five classes on the wealth index: poorest, poorer, middle, richer, and richest [19]. We used the existing coding in the DHS for internet usage, sex of household head, and place of residence. We segregated the variables into individual level and contextual levels regarding literature that used the DHS dataset to examine health insurance coverage [11, 14, 15, 23, 24]. The categories of the variables can be found in Table 1.

### Statistical analyses

We used Stata software, version 17.0 (Stata Corporation, College Station, TX, USA) for all the analyses. We used percentages to present the proportion of women who were covered by health insurance. Later, using cross-tabulation, we examined how health insurance coverage was distributed among the explanatory factors. Also, a Pearson chi-square test of independence was used to determine the explanatory variables significantly associated with health insurance coverage at *p* < 0.05. Before the regression analysis, we checked for possible collinearity among the studied variables using the variance inflation factor (VIF). The results showed that the minimum, maximum, and mean VIFs were 1.04, 4.10, and 1.89, respectively. Hence, there was no evidence of multicollinearity among the variables. We used the multilevel binary logistic analysis to examine the factors associated with health insurance coverage. Multilevel regression analysis was adopted for the study due to the complex design used in the DHS involving a two-stage cluster sampling. We used four models to examine the factors. Model O was an empty model with no explanatory variables. Model I, Model II, and Model III included the individual level, contextual level, and all explanatory variables, respectively. We presented the results adjusted odds ratio (aOR) with their respective 95% confidence intervals (CIs). In addition, all four models included fixed and random effects. Random effects denoted the measure of variation in the health insurance coverage based on primary sampling unit (PSU) measured by Intra-Cluster Correlation Coefficient [ICC], whereas fixed effects denoted the relationship between the explanatory variables and the outcome variable. Model fitness, or how well different models match the data, was assessed using Akaike Information Criterion (AIC). The “melogit” program in Stata was used to execute the multilevel regression models. Statistical significance was set at *p* < 0.05. We also weighted all the analyses to adjust for disproportionate sampling and non-response.

### Ethical consideration

Ethical clearance was not sought for this study since we analyzed secondary data already available in the public domain. We sought permission for the Monitoring and Evaluation to Assess and Use Results Demographic and Health Surveys (MEASURE DHS) and it was granted before using the dataset.

## Results

### Prevalence of health insurance coverage among the women

**Fig. 1:**
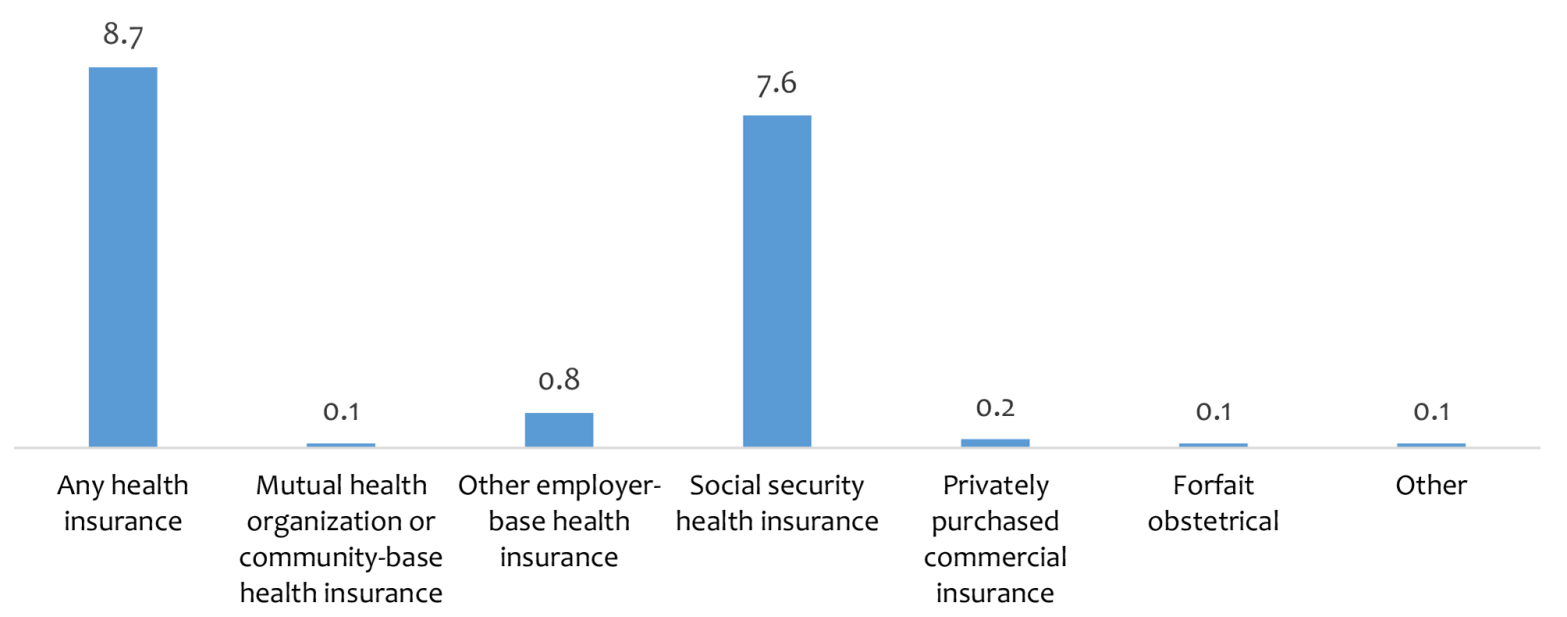
Coverage of health insurance

Overall, the coverage of health insurance among the women was 8.7%. Of this proportion of women, the majority subscribed to social security health insurance (7.6%) followed by employer-based health insurance (0.8%) as shown in Fig. 1.

### Distribution of health insurance coverage across the explanatory variables

Table 1 presents the results of the distribution of health insurance coverage across the explanatory variables. The results showed that health insurance coverage is prevalent among women aged 35 and above (11.6%), those who had attained higher education (35.6%), those who were working (12.3%), those with multiparity (9.8%), and those exposed to mass media: television (14.1%), radio (10.9%), newspaper or magazine (17.5%), and internet (16.9%). Additionally, the percentage of health insurance coverage was high among women with male household heads (9.3%), those in the richest wealth index (25.7%), those in the urban areas (15.3), and those from Tiris zemour et Inchiri region (31.5%). Except for marital status, all the explanatory variables were statistically associated with health insurance coverage at *p* < 0.05.

**Table 1:** Distribution of health insurance coverage across explanatory variables

Variable	Weighted		Health insurance subscription	
	Sample	Percentage	Yes (%)	***P***-value
**Women’s age (years)**				< 0.001
15–24	6,403	40.7	7.5 [6.3, 9.0]	
25–34	4,816	30.7	7.7 [6.6, 8.9]	
35 and above	4,495	28.6	11.6 [10.2, 13.1]	
**Level of education**				< 0.001
No education	5,153	32.8	2.3 [1.8, 3.0]	
Primary	6,056	38.5	5.0 [4.2, 5.9]	
Secondary	4,144	26.4	19.9 [17.4, 22.6]	
Higher	361	2.3	35.6 [29.9, 41.7]	
**Current working status**				< 0.001
Not working	12,552	79.9	7.8 [6.8, 8.9]	
Working	3,162	20.1	12.3 [10.4, 14.6]	
**Marital status**				0.960
Never in union	4,185	26.6	8.9 [7.5, 10.5]	
Married	9,825	62.5	8.7 [7.7, 9.8]	
Widowed	228	1.5	9.5 [5.6, 15.9]	
Divorced	1,476	9.4	8.5 [6.4, 11.0]	
**Parity**				0.031
Nulliparity	5,666	36.0	8.9 [7.5, 10.5]	
Primiparity	1,888	12.0	7.5 [5.9, 9.5]	
Multiparity	4,489	28.6	9.8 [8.6, 11.1]	
Grand parity	3,671	23.4	7.8 [6.7, 9.1]	
**Watch television**				< 0.001
No	7,436	47.3	2.7 [2.0, 3.6]	
Yes	8,278	52.7	14.1 [12.4, 16.0]	
**Listen to radio**				< 0.001
No	8,736	55.6	7.0 [6.0, 8.2]	
Yes	6,978	44.4	10.9 [9.5, 12.4]	
**Read newspapers or magazines**				< 0.001
No	13,095	83.3	7.0 [6.0, 8.1]	
Yes	2,619	16.7	17.5 [15.1, 20.2]	
**Internet usage**				< 0.001
No	9,916	63.1	4.0 [3.4, 4.6]	
Yes	5,798	36.9	16.9 [15.0, 19.0]	
**Sex of household head**				0.038
Male	9,367	59.6	9.3 [8.1, 10.7]	
Female	6,347	40.4	7.9 [6.7, 9.2]	
**Wealth index**				< 0.001
Poorest	2,706	17.2	0.3 [0.2, 0.6]	
Poorer	2,854	18.2	0.9 [0.6, 1.4]	
Middle	3,145	20.0	3.6 [2.7, 4.8]	
Richer	3,474	22.1	9.1 [7.6, 10.8]	
Richest	3,535	22.5	25.7 [22.9, 28.7]	
**Place of residence**				< 0.001
Urban	8,053	51.2	15.3 [13.4, 17.4]	
Rural	7,661	48.8	1.8 [1.4, 2.3]	
**Region**				< 0.001
Hodh Echargui	2,034	12.9	1.7 [1.0, 2.9]	
Hodh Gharbi	1,579	10.1	4.2 [2.0, 8.8]	
Assaba	1,249	8.0	3.8 [2.6, 5.5]	
Gorgol	1,293	8.2	4.0 [2.3, 6.8]	
Brakna	1,282	8.2	4.8 [3.3, 6.8]	
Trarza	961	6.1	7.0 [5.0, 9.6]	
Adrar	298	1.9	12.8 [10.1, 16.1]	
Dakhlet Nouadhibou	539	3.4	22.2 [15.4, 30.9]	
Tagant	349	2.2	8.0 [5.2, 12.3]	
Guidimagha	1,244	7.9	1.5 [0.7, 2.9]	
Tiris zemour et Inchiri	273	1.7	31.5 [22.7, 41.9]	
Nouakchott Ouest	793	5.1	15.3 [10.6, 21.5]	
Nouakchott Nord	2,073	13.2	17.2 [13.3, 21.8]	
Nouakchott Sud	1,747	11.1	15.9 [11.2, 22.0]	

### Factors associated with health insurance coverage among the women

#### Fixed effect results

Table 2, Model III presents the factors associated with health insurance coverage among women in Mauritania. Women aged 35 years and above were more likely to be covered by health insurance compared to those aged 15–24 [aOR = 1.54; 95% CI = 1.24, 1.92]. The likelihood of being covered by health insurance increased with increasing level of education with the highest odds among women with higher education [aOR = 6.09; 95% CI = 3.93, 9.42]. Similarly, the odds of health insurance coverage increase with increasing parity, with the highest odds among those with grand parity [aOR = 2.16; 95% CI = 1.62, 2.87]. Additionally, women who were working [aOR = 1.42; 95%CI = 1.15, 1.76], those exposed to watching television [aOR = 1.42; 95%CI = 1.07, 1.89], and those who used the internet [aOR = 1.37; 95% CI = 1.12, 1.67] were more likely to be covered by health insurance compared to their counterparts who were not working, did not watch television, and did not use internet, respectively. Moreover, compared with women in the poorest wealth index, those in the richest wealth quintile had the highest odds of being covered by health insurance [aOR = 22.12; 95% CI = 9.52, 51.41]. We found that women residing in Tiris zemour et Inchiri [aOR = 3.60; 95%CI = 1.60, 8.10], Tagant (aOR = 3.74; 95% CI = 1.61, 8.68], and Adrar [aOR = 2.76; 95% CI = 1.36, 5.61] regions were more likely to be covered by health insurance compared with those from Hodh Echargui.

#### Random effects results

Table 2 presents the results of the random effect. We found that the value of ICC in Model O was 0.464, which shows that about 46.4% of the variation in health insurance coverage is attributable to the variations between the clusters. The variation between clusters indicated by the ICC values decreased from 0.228 in Model I to 0.137 in Model III, the model with all the explanatory variables. Additionally, we chose Model III and interpreted its results for discussion because it’s the model with the least AIC value (6798.065) compared to the other models affirming the goodness of fit.

**Table 2:** Factors associated with health insurance subscription among women in Mauritania

Variable	Model O	Model I aOR [95% CI]	Model II aOR [95% CI]	Model III aOR [95% CI]
**Fixed effect results**				
**Women’s age (years)**				
15–24		1.00		1.00
25–34		0.93 [0.76, 1.13]		0.88 [0.72, 1.07]
35 and above		1.92^***^ [1.56, 2.37]		1.54^***^ [1.24, 1.92]
**Level of education**				
No education		1.00		1.00
Primary		2.04^***^ [1.47, 2.82]		1.75^***^ [1.27, 2.42]
Secondary		6.62^***^ [4.62, 9.47]		4.51^***^ [3.17, 6.40]
Higher		9.60^***^ [6.09, 15.13]		6.09^***^ [3.93, 9.42]
**Current working status**				
Not working		1.00		1.00
Working		1.37^**^ [1.11, 1.70]		1.42^**^ [1.15, 1.76]
**Parity**				
Nulliparity		1.00		1.00
Primiparity		0.96 [0.70, 1.32]		0.98 [0.72, 1.34]
Multiparity		1.63^***^ [1.29, 2.07]		1.73^***^ [1.35, 2.21]
Grand parity		2.00^***^ [1.52, 2.63]		2.16^***^ [1.62, 2.87]
**Read newspapers or magazines**				
No		1.00		1.00
Yes		1.24 [0.98, 1.56]		1.24 [0.98, 1.56]
** Listen to the radio**				
No		1.00		1.00
Yes		0.93 [0.79, 1.09]		0.97 [0.82, 1.14]
** Watch television**				
No		1.00		1.00
Yes		2.16^***^ [1.56, 2.99]		1.42^*^ [1.07, 1.89]
**Internet usage**				
No		1.00		1.00
Yes		1.89^***^ [1.54, 2.32]		1.37^**^ [1.12, 1.67]
**Sex of household head**				
Male			1.00	1.00
Female			0.89 [0.75, 1.05]	0.87 [0.72, 1.04]
**Wealth index**				
Poorest			1.00	1.00
Poorer			2.43^*^ [1.11, 5.31]	2.09 [0.96, 4.57]
Middle			7.44^***^ [3.45, 16.04]	5.07^***^ [2.30, 11.19]
Richer			16.23^***^ [7.48, 35.23]	8.90^***^ [3.92, 20.18]
Richest			54.28^***^ [24.71, 119.24]	22.12^***^ [9.52, 51.41]
**Place of residence**				
Urban			1.00	1.00
Rural			0.52^***^ [0.36, 0.75]	0.71 [0.49, 1.03]
**Region**				
Hodh Echargui			1.00	1.00
Hodh Gharbi			1.60 [0.71, 3.60]	1.32 [0.60, 2.91]
Assaba			1.62 [0.72, 3.67]	1.63 [0.74, 3.60]
Gorgol			1.68 [0.76, 3.75]	1.41 [0.63, 3.14]
Brakna			1.78 [0.89, 3.59]	1.80 [0.89, 3.65]
Trarza			1.91 [0.93, 3.95]	1.78 [0.86, 3.68]
Adrar			2.88^**^ [1.39, 5.96]	2.76^**^ [1.36, 5.61]
Dakhlet Nouadhibou			1.53 [0.71, 3.33]	1.63 [0.73, 3.61]
Tagant			4.22^***^ [1.85, 9.64]	3.74^**^ [1.61, 8.68]
Guidimagha			0.69 [0.28, 1.75]	0.72 [0.30, 1.73]
Tiris zemour et Inchiri			3.85^***^ [1.73, 8.59]	3.60^**^ [1.60, 8.10]
Nouakchott Ouest			1.37 [0.62, 3.03]	1.24 [0.56, 2.74]
Nouakchott Nord			1.81 [0.87, 3.78]	1.62 [0.77, 3.42]
Nouakchott Sud			1.37 [0.64, 2.93]	1.39 [0.64, 3.01]
**Random effect model**				
PSU variance (95% CI)	2.851 [2.271, 3.580]	0.972 [0.688, 1.373]	0.550 [0.392, 0.773]	0.523 [0.374, 0.732]
ICC	0.464	0.228	0.143	0.137
Wald chi-square	Reference	497.70 (< 0.001)	682.09 (< 0.001)	1098.08 (< 0.001)
**Model fitness**				
Log-likelihood	-3990.6507	-3557.7908	-3603.8833	-3365.0327
AIC	7985.301	7145.582	7249.767	6798.065
BIC	8000.626	7260.516	7410.675	7058.584
N	15,714	15,714	15,714	15,714
Number of clusters	406	406	406	406

aOR = adjusted odds ratios; CI = Confidence Interval; ^*^p < 0.05, ^**^p < 0.01, ^***^p < 0.001; 1.00 = Reference category; PSU = Primary Sampling Unit; ICC = Intra-Class Correlation Coefficient; AIC = Akaike Information Criterion; BIC = Bayesian Information Criterion

## Discussion

We examined the coverage and determinants of health insurance among Mauritanian women. We found that the coverage of health insurance to be 8.7%. Factors identified to be associated with health insurance coverage were age, level of education, current working status, parity, exposure to watching television, internet usage, wealth index, and region.

Our finding showed that the coverage of health insurance among women in Mauritania is low (8.7%). This finding contradicts that of Ghana [14] but is similar to that of Tanzania which recorded health insurance coverage of 62.4% and 9.1%, respectively. The disparities in health insurance coverage between the countries could be due to the health policy priority, sociocultural, and demographic variation in these countries. Our result is consistent with health insurance coverage from studies conducted in Mauritania [25], Ethiopia [26], and Nigeria [27] where the coverage was less than 10% among women in those countries. The low health insurance coverage found in this study could have negative implications for the attainment of the SDG target 3.8 if not addressed holistically. The low health insurance coverage among women as found in this current study is ascribed to the household inequality between men and women in the control and access to the household financial resources and also decision-making about health choices. Women in many households across the global south of which Mauritanian is no exception have limited access to household financial resources as well as a reduced role in health decision-making. Therefore, these women might be less likely to decide on their own volition to subscribe to health insurance without the consent of their husbands who can eventually refuse a such suggestion. This observation points to the need for the government and national health authorities in Mauritania to programmes that will empower women to own their health and be able to make health decisions without impediment from spouses. The low health insurance coverage could stem from issues such as ineffective procedures for collecting premiums, poor understanding of rules, misinformation by recruiters and mistrust among the populace and the health insurance organizations, and low awareness and attitudinal problems of the populace towards health insurance.

Women aged 35 years and above were more likely to subscribe to health insurance than their younger counterparts. The results concur with literature reviews conducted in West Africa [28] and Nigeria [27] which suggest that people who are 30 years or older have a higher likelihood of subscribing to health insurance. This could be attributed to the fact that people of advanced age relatively have a higher probability of falling sick as their health deteriorates with aging, hence their decision to subscribe to health insurance as they are likely to have frequent health utilization. These findings could also be because people who are 35 years or older could be economically active and hence have purchasing power to pay for their health insurance subscriptions and premiums. On the other hand, older women may be mothers and/or caregivers who are more likely to have more children who may often fall sick hence their decision to subscribe to health insurance to prevent out-of-pocket payment. Women in this category as well may have gone through childbearing and are well abreast with the expensive medical bills one will have to pay during pregnancy, childbirth, and postnatal care. This could also explain the higher odds of health insurance coverage among women who had given birth before, especially, those with multiparity and grand parity.

Educated women were more likely to subscribe to health insurance in this study than those without any form of education. Our results are similar to findings reported in Ghana [29, 30], Peru [31], Zambia [32], and SSA [14, 15] where educated women were more likely to be covered by health insurance. Educated women are more likely to be employed and financially capable of paying subscription and premium fees. They are also well-informed and able to access health information about health which may drive them to hold positive health-seeking behaviors. In addition, educated women are likely to have their health insurance covered by their employers since many educated individuals tend to be employed. As with Ghana, workers have health insurance deducted from their Social Security and National Insurance Trust (SSNIT) contributions and only pay a small upfront fee to be covered [33].

In furtherance, women who were employed or currently working had higher odds of being covered by insurance. Our findings corroborate with studies conducted in Malaysia [34], East Africa [5], and Ghana [17], where employed women were more likely to be covered by health insurance. This is closely associated with women’s ability to pay for subscriptions and renewal of premiums as well as being supported by employers in this regard. The results show that when women are empowered financially, they can take care of their health needs by subscribing to insurance policies that will prevent catastrophic out-of-pocket payments.

We found exposure to mass media: watching television and internet usage to be associated with health insurance coverage. These findings strongly agree with studies conducted in East Africa [5], Rwanda [35], Ethiopia [26], and Zambia [32] where accessibility and usage of print and electronic media increase the odds of subscribing to health insurance. Our finding on internet usage is similar to the association of health insurance coverage in Ethiopian women [26]. Access to mass, and watch adverts and health educational programs that emphasize the need to be covered by health insurance to prevent out-of-pocket payments. These pieces of information are also important for women to make critical decisions about their health. To meet the universal health coverage goal of 2030, various national health departments and ministries have had to implement health insurance as a pro-poor initiative to reduce household expenditure on health. As such, deliberate media campaigns can be pivotal in awareness creation and sensitization for subscription to health insurance.

Health insurance coverage was associated with women’s household wealth index. Health insurance is meant to cushion households from the severe catastrophic burden of health financing. Yet, those in much poorer homes are less likely to subscribe [36]. Increasing wealth index showed higher odds of women being covered by health insurance in this study, which confirm the findings of several studies conducted across the world [5, 24, 29, 35]. These findings reflect the need for women’s empowerment, health utilization, and promotion of maternal and child health. Empowered women can break the power dynamics and have provident decisions and control over their health and that of their children. Current results contradict the high subscription of health insurance among core poor Ghanaians [33]. High rates of poor enrolment in health insurance schemes are related to pro-poor interventions such as Livelihood Empowerment Against Poverty (LEAP) which provides financial support to the poor to be able to subscribe to health insurance and exemptions for the elderly, and children, subsidized subscription for pregnant women among others.

The geographical region of residence was found to be associated with health insurance coverage. Specifically, women from Tagant, Tiris zemour et Inchiri, and Adrar regions were more likely to be covered by health insurance. Prior studies have also revealed different levels of health insurance coverage in regions of countries such as Malawi, Nigeria, Ghana, Kenya, and Ethiopia [3, 26, 27, 29, 37]. Factors such as high literacy and availability of community-based insurance schemes could have contributed to the higher likelihood of health insurance coverage among women residing in the aforementioned regions in Mauritania. In our current study, the identified regions could be highly associated with increased literacy rates giving the populace in these areas access to information about health insurance hence their high subscription.

### Public health policy implication

The low health insurance coverage could lead to poor health service utilization among women in Mauritania. Targeted education and awareness creation across the country to whip up interest in health insurance subscriptions is highly recommended. Women’s empowerment strategies in the form of employment, education, and access to information among others could serve as enabling environments for increased subscription. By emphasizing the advantages and significance of coverage, policymakers should concentrate on raising health insurance awareness among women in Mauritania. Additionally, the relationship between wealth index and health insurance enrollment draws attention to the socioeconomic coverage gaps. As a result, efforts should be taken to guarantee that health insurance procedures and rates are fair and reasonable for those in households with lower wealth indices. To facilitate enrollment and lower financial barriers in communities, this can entail targeted subsidies or financial assistance programs.

### Strength and limitation

The current study used the most recent nationwide dataset Additionally, our study is the first of its kind to the best of our study knowledge to be conducted among women in Mauritania. Some limitations need to be acknowledged. Since the data analyzed was secondary sourced, there may be other factors or variables that could have influenced health insurance coverage which were not included in the study because they were not available in the DHS dataset. Also, the cross-sectional nature of the DHS limits the study’s ability to make causal inferences. Additionally, data was collected using self-reports. Hence, the data may be prone to recall and social desirability biases.

## Conclusion

Health insurance coverage among Mauritanian women was low in this study. With less than a tenth, subscription to insurance highlights potential key problems to the country’s journey to the realization of universal primary health access without the cost being an impediment. We recommend effective public education and awareness creation on the importance of being covered by health insurance by leveraging television and internet platforms. Also, interventions to increase health insurance coverage should consider younger women and those in rural areas. Also, achieving the SDG targets of ensuring universal health coverage and lowering maternal mortality to less than 70 deaths per 100,000 live births requires the implementation of interventions to increase health insurance coverage, taking into consideration the identified factors in the study.

## Data Availability

The dataset used can be accessed via the MEASURE DHS repository https://dhsprogram.com/data/dataset/Mauritania_Standard-DHS_2020.cfm?flag=1.

## Abbreviations

AIC: Akaike Information Criterion
aOR: Adjusted Odds Ratio
CIs: Confidence Intervals
DHS: Demographic and Health Survey
LMICs: Low-and middle-income countries
SDG: Sustainable Development Goal
SSA: sub-Saharan Africa
STROBE: Strengthening the Reporting of Observational Studies in Epidemiology
UHC: Universal Health Coverage
WHO: World Health Organization
